# Distinct gene expression dynamics in germ line and somatic tissue during ovariole morphogenesis in *Drosophila melanogaster*

**DOI:** 10.1093/g3journal/jkab305

**Published:** 2021-09-06

**Authors:** Shreeharsha Tarikere, Guillem Ylla, Cassandra G Extavour

**Affiliations:** 1 Department of Organismic and Evolutionary Biology, Harvard University, Cambridge, MA 02138, USA; 2 Department of Molecular and Cellular Biology, Harvard University, Cambridge, MA 02138, USA; 3 Howard Hughes Medical Institute, Chevy Chase, MD 20815, USA

**Keywords:** ovary, FACS, RNA-seq, terminal filament, germ line, stem cell niche

## Abstract

The survival and evolution of a species is a function of the number of offspring it can produce. In insects, the number of eggs that an ovary can produce is a major determinant of reproductive capacity. Insect ovaries are made up of tubular egg-producing subunits called ovarioles, whose number largely determines the number of eggs that can be potentially laid. Ovariole number in *Drosophila* is directly determined by the number of cellular structures called terminal filaments, which are stacks of cells that assemble in the larval ovary. Elucidating the developmental and regulatory mechanisms of terminal filament formation is thus key to understanding the regulation of insect reproduction through ovariole number regulation. We systematically measured mRNA expression of all cells in the larval ovary at the beginning, middle, and end of terminal filament formation. We also separated somatic and germ line cells during these stages and assessed their tissue-specific gene expression during larval ovary development. We found that the number of differentially expressed somatic genes is highest during the late stages of terminal filament formation and includes many signaling pathways that govern ovary development. We also show that germ line tissue, in contrast, shows greater differential expression during early stages of terminal filament formation, and highly expressed germ line genes at these stages largely control cell division and DNA repair. We provide a tissue-specific and temporal transcriptomic dataset of gene expression in the developing larval ovary as a resource to study insect reproduction.

## Introduction 

Healthy reproductive organs are among the most important factors that determine the fertility of an individual, and more importantly, continuity of the species itself. Reproductive fitness, including fecundity, is determined by the number of progenies an organism can produce. In insects, egg-producing subunits of ovaries are called ovarioles (Büning 1994). In flies of the genus *Drosophila*, the number of ovarioles predicts the peak egg-laying potential of the females of the species (David 1970), and is negatively correlated with egg size but positively correlated with reproductive output (Church *et al.* 2021). The number of ovarioles varies widely across insects and is in the range of 18–24 ovarioles per ovary in wild type North American populations of *Drosophila melanogaster* (Honěk and Honek 1993; Markow and O'Grady 2007; Hodin 2009). In *Drosophila*, adult ovariole number is established in the larval stages through the development of a species-specific number of linear somatic cell stacks called terminal filaments (King *et al.* 1968). The number of terminal filaments assembled by the time of pupariation usually predicts adult ovariole number (King 1970; Hodin and Riddiford 2000). Thus, terminal filaments are the primordial larval structures whose number ultimately determines the ovariole number. The genetic mechanisms governing ovary morphogenesis, which includes the process of regulation of terminal filament number and assembly during larval ovary development, remain poorly understood.

**Figure 1:**
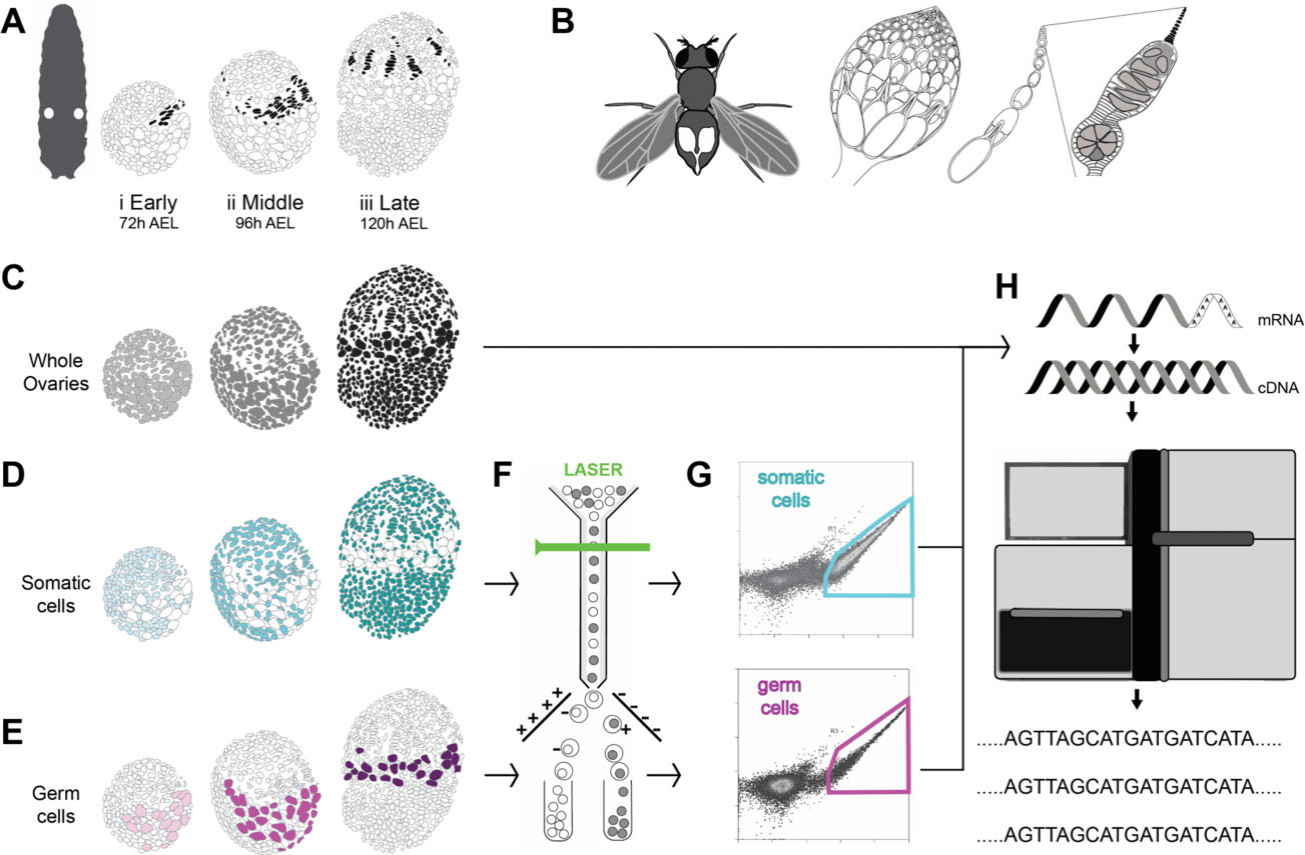
Experimental scheme for generating stage-specific transcriptomes of germ cells and somatic cells of larval ovaries during terminal filament formation. (A) Location of the larval ovaries (white circles within the larva), and illustration of larval ovary development divided into three stages during terminal filament formation (colored in black). (B) Left to right: location of the ovaries in an adult female abdomen; a single adult ovary containing multiple ovarioles; an individual ovariole; anterior tip of an ovariole enlarged to show the germarium and terminal filament (black) at the tip. (C) Representation of the three stages of whole larval ovaries from the wild type strain Oregon R, chosen for library preparation and sequencing (light gray: early stage, gray: mid, dark gray: late). (D) Somatic cells and (E) germ cells from developing ovaries at the three chosen stages were labeled with GFP using tissue-specific GAL4 lines (somatic cells in shades of cyan and germ cells in shades of magenta). Somatic cells were labeled using *bab: GAL4* (genotype: w[*]; P{w[+mW.hs]=GawB}bab1[Pgal4-2]/TM6B, Tb[1]) and germ cells were labeled using *nos: GAL4* (genotype: *P{w[+mC]=UAS-Dcr-2.D}1, w[1118]; P{w[+mC]=GAL4-nos.NGT}40).* (F) GFP-positive cells were separated using Flourescence-activated cell sorting. (G) Schematics of representative plot layouts of somatic and germ line tissue separation using FACS. Y-axis: autofluorescence, 488-576/21 Height Log; X-axis: GFP fluorescence intensity, 488-513/26 Height Log (see Supplementary Figure S2 for actual representative data plots). (H) Separated cells or whole ovaries were processed for mRNA extraction and cDNA library preparation followed by high throughput sequencing. h AEL = hours After Egg Laying.

Ovary morphogenesis is orchestrated by interactions of the cell types of somatic and germ line tissues. Larval somatic ovarian tissue is principally made up of five cell types—sheath cells, swarm cells, terminal filaments, cap cells, and intermingled cells. The anteriormost cells of the ovary are the sheath cells, and a sub-population of these apically positioned cells undergo two cell migration events during larval ovary development. First, a population of sheath cells called swarm cells migrates from the anterior to the posterior of the ovary to form the basal region in the mid third larval instar stage (Couderc *et al.* 2002; Green and Extavour 2012). Second, in the late third instar and early pupal stages, sheath cells migrate from the apical to the basal region, traversing in between terminal filament cells (King *et al.* 1968). These sheath cells lay down basement membrane in their path, which encapsulates developing ovarioles (King 1970). Terminal filaments are stacks of cells located just below the sheath cells in the anterior larval ovary. They are formed by a process of progressive intercalation of flattened cells into stacks, and stack formation occurs in a “wave” that proceeds from the medial to the lateral side in the larval ovary (Figure 1A; Sahut-Barnola *et al.* 1995).

The genes *bric à brac 1 (bab1)* and *bric à brac 2 (bab2)* are expressed in the terminal filaments and essential for terminal filament cell differentiation and terminal filament assembly (Godt and Laski 1995; Sahut-Barnola *et al.* 1995; Couderc *et al.* 2002; Saler *et al.* 2020). The gene *engrailed* is also expressed in terminal filaments, and at a lower level in the cap cells of the larval ovary (Bolivar *et al.* 2006; Saler *et al.* 2020). Clones homozygous for an *engrailed* deletion allele generated in terminal filaments in third instar larvae showed that this gene is required in initial terminal filament precursors for the correct assembly of terminal filaments. (Bolivar *et al.* 2006). However, a subsequent study showed that RNAi knockdown of *engrailed* and *invected* driven by *bab: GAL4* in larval terminal filament and cap cells does not affect terminal filament formation (Saler *et al.* 2020). This could mean that *engrailed/invected* are not absolutely required for terminal filament formation, but that genetic heterogeneity with respect to *engrailed/invected* dose, is important among terminal filament precursor cells to ensure correct terminal filament morphogenesis. Accumulation of *engrailed* in terminal filaments is dependent on *bab* gene expression (Saler *et al.* 2020).

We previously showed that the *Hippo* signaling pathway controls the regulation of cell proliferation in somatic cells, thereby affecting the number of terminal filaments and their constituent terminal filament cells (Sarikaya and Extavour 2015). During early terminal filament formation, Actin and Armadillo (Arm) proteins deposited in the region between terminal filaments make a scaffold to flatten and intercalate terminal filament cells (Godt and Laski 1995; Sahut-Barnola *et al.* 1995; Chen *et al.* 2001). Expression of the protein cofilin (*twinstar*) is required in terminal filament and apical cells for actin-based change in cell shape, and loss of cofilin causes a reduction in terminal filament and apical cell numbers (Chen *et al.* 2001).

Normal growth of an ovary depends on the homeostatic proliferation of the somatic and germ line tissues (Gilboa and Lehmann 2006; Gilboa 2015). This balance between somatic and germ line tissue populations is achieved by regulation of proliferation, differentiation, and apoptosis of stem cell populations of somatic and germ cell lineages (Sahut-Barnola *et al.*^1995^, ^1996^). Somatic cells called intermingled cells interact with the germ cells and control their proliferation (Li *et al.* 2003, 2019; Gilboa and Lehmann 2006; Sarikaya and Extavour 2015; Lai *et al.* 2017; Panchal *et al.* 2017). *Notch, hedgehog, Mitogen Activated Protein Kinase (MAPK)* and *Epidermal growth factor receptor* (*EGFR)* signaling pathways, as well as the transcription factor *traffic jam*, maintain the germ line stem cell niche (Besse *et al.* 2005; Song *et al.* 2007; Matsuoka *et al.* 2013; Sarikaya and Extavour 2015; Yatsenko and Shcherbata 2021), which is established at the base (posterior) of each terminal filament.

Recent work by Slaidina and colleagues used single-cell transcriptomics to describe the gene expression profiles of the various cell types of the late third instar larval ovary (Slaidina *et al.* 2020). They sub-divided terminal filament cells into anterior or posterior cell types, and sheath cells into migratory or nonmigratory cell types, based on gene expression patterns of the single cell sub-populations. While this study examined a single time point of ovary development, given that ovary morphogenesis is a temporal process, we hypothesize that changes in gene expression patterns over the course of development may be important to regulate morphogenesis. Thus, a gene expression study across the developing stages of larval ovary would advance our understanding of the transcriptomic regulation of ovarian morphogenesis.

Although all major conserved animal signaling pathways are known to be involved in ovarian morphogenesis (Twombly *et al.* 1996; Cohen *et al.* 2002; Huang *et al.* 2005; Song *et al.* 2007; Gancz and Gilboa 2013; Green and Extavour 2014; Sarikaya and Extavour 2015; Kumar *et al.* 2020), a systematic gene expression profile of a developing ovary is lacking. Such system-wide gene expression data for the ovary throughout terminal filament morphogenesis, including the potentially distinct transcriptional profiles of germ cells and somatic cells, could shed light on the processes involved in the maintenance of cell types necessary to shape the ovary and control the number of ovarioles.

To this end, we measured gene expression during the development of the larval ovary by systematically staging and sequencing mRNA from whole ovaries before, during, and after terminal filament formation. Furthermore, we separated somatic and germ line tissue types at each of these stages to analyze tissue-specific gene expression. We compared the gene expression profiles across tissues and also across stages of ovary development. We then employed functional enrichment analysis to determine the different biological functions active in the three larval developmental stages and two tissue types that could yield information on ovary morphogenesis. This dataset is an important temporal and tissue specific gene expression resource for the insect developmental biology community to understand early ovary development.

## Materials and methods

### Fly stocks

Flies were reared at 25°C at 60% humidity with food containing yeast and in uncrowded conditions. The following two fly lines were obtained from the Bloomington *Drosophila* Stock Center: w[*]; P{bab1[Pgal^4-2^]/TM6B, Tb[1] (abbreviated herein as *bab: GAL4*; stock number 6803), *P{w[+mC]=UAS-Dcr-2.D}1, w[1118]; P{w[+mC]=GAL4-nos.NGT}40* (abbreviated herein as *nos: GAL4*; stock number 25751). *w[1118], P[UAS Stinger]* (abbreviated herein as *UAS: Green Stinger I*, Barolo *et al.* 2000) used for GFP expression was a gift from Dr. James Posakony (University of California, San Diego). Crosses were set with 100–200 virgin UAS females and 50–100 GAL4 males in a 180 ml bottle containing 50 ml standard fly media 1 day prior to egg laying.

### Staging larvae

To obtain uniformly staged larvae for the experiments, a protocol was devised to collect eggs that were near-synchronously laid, from which the larvae were then collected. To obtain a desired genotype, crosses were set as described above. The cross was set at 25°C at 60% humidity and left overnight to mate. Hourly egg collections were set up on 60 mm apple juice-agar plates (9 g agar, 10 g sugar, 100 ml apple juice, and 300 ml water) with a pea-sized spread of fresh yeast paste (baker’s yeast granules made into a paste in a drop of tap water). Eggs were collected hourly for 8 h. The first two collection plates were discarded to remove asynchronously laid eggs that may have been retained inside the females following fertilization. Staged first instar larvae were collected into vials 24 h after egg collection. Larvae at 72 h AEL (hours After Egg Laying) were designated as early stage, at 96 h AEL as mid stage and at 120 h AEL as late stage of Terminal Filament development. For a step-by-step detailed protocol see Supplementary File S1.

### Dissection and dissociation of larval ovary

Staged larvae were collected for dissection every hour. The head of the larva was removed with forceps and the cuticle and gut were carefully pulled with one forceps while holding the fat body with another forceps. This process left just the fat bodies in the dissection dish as long as the larvae were well fed and fattened with yeast. Ovaries located in the center of the length of each fat body were then dissected free of the fat body using an insulin syringe needle (BD 328418). Ovaries dissected clear of fat body were collected in DPBS (Thermo Fisher 14190144) and batches of 20–30 ovaries in DPBS were kept on ice until dissociation. Ovaries were harvested hourly at the appropriate times, placed on ice immediately following dissection, and maintained on ice for a maximum of 4 h before dissociation and subsequent FACS processing.

Dissociation of the larval ovary required two enzymatic steps. After 7 h of dissection, all the dissected ovaries were placed in 0.25% Trypsin solution (Thermo Fisher 25200056) for 10 min at room temperature in the cavity of a glass spot plate (Fisher Scientific 13-748B). They were then transferred to another cavity containing 2.5% Liberase (5 g Liberase reconstituted in 2 ml nuclease free water; Sigma 5401119001) and teased apart with insulin syringe needles until most of the clumps were separated and left (without agitation) at room temperature for 10 min. Using a 200 µl pipette with a filter tip (pre-rinsed in 1X PBS), the dissociated cells in Liberase were pipetted up and down gently ten times to uniformly mix and separate the cells. The cell suspension was then transferred to an RNA Lobind tube (Eppendorf 8077-230) and placed on a vortexer for 1 min. Meanwhile, the well was rinsed in 1.4 ml of PBS by pipetting repeatedly. This PBS was then mixed with the cell-suspension in Liberase and vortexed for another minute, and the entire sample was then placed on ice. This sample was then taken directly to the FACS facility on ice along with an RNA Lobind collection tube containing 100–200 µl Trizol (Thermo Fisher 15596026). For a step-by-step detailed protocol see Supplementary File S1.

### Flow sorting GFP-positive cells

The dissociated tissue sample was sorted in a MoFlo Astrios EQ Cell sorter (Beckman Coulter) run with Summit v6.3.1 software. The dissociated cell solution was diluted and a flow rate of 200 events per second was maintained with high sorting efficiency (<98%) during the sorting process. A scatter gate (R1) was employed to eliminate debris (Supplementary Figure S2) and a doublet gate (R2) was used to exclude nonsinglet cells. A 488 nm emission Laser was used to excite the GFP, and the collection was at 576 nm. The GFP-positive cells were designated in gate R3 and sorted directly into Trizol. The resulting cells collected in Trizol were frozen immediately by plunging the tube into liquid nitrogen and then stored at −80°C until RNA extraction. A single replicate consisted of at least 1000 cell counts pooled from FACS runs.

### RNA extraction

Flow-sorted cells, stored at −80°C were thawed at room temperature. Trizol contents were lysed with a motorized pellet pestle (Kimble 749540-0000). Zymo RNA Micro-Prep kit (Zymo Research R2060) was used to isolate RNA from the Trizol preparations. Equal amounts of molecular grade ethanol (Sigma E7023) were added to Trizol and mixed well with a pellet pestle, then pipetted onto a spin column. All centrifugation steps were done at 10,000 g for one minute at room temperature. The column was washed with 400 µl Zymo RNA wash buffer and then treated with Zymo DNase (6U/µl) for 15 min at room temperature. The column was then washed twice with 400 µl Zymo RNA Pre-wash buffer and once with Zymo RNA wash-buffer. The RNA was eluted from the column in 55 µl of Nuclease-free water (Thermo Fisher 10977015). The RNA obtained was quantified first using a NanoDrop (Model ND1000) spectrophotometer and then using a high sensitivity kit (Thermo Fisher Q32852) on a Qubit 3.0 Fluorometer (Thermo Fisher Q33216).The extracted RNA was also checked for integrity on a high sensitivity tape (Agilent 5067-5579) with an electronic ladder on an Agilent Tapestation 2200 or 4200. RNA extraction from staged whole ovaries was carried out by crushing entire ovaries in Trizol and following the same protocol described above. For a step-by-step detailed protocol see Supplementary File S1.

### Library preparation

cDNA libraries were prepared using the Takara Apollo library preparation kit (catalogue # 640096). Extracted RNA samples were checked for quality using Tapestation tapes. Fifty microliters of RNA samples were pipetted into Axygen PCR 8-strip tubes (Fisher Scientific 14-222-252) and processed through PrepX protocols on the Apollo liquid handling system. mRNA was isolated using PrepX PolyA-8 protocol (Takara 640098). The mRNA samples were then processed for cDNA preparation using PrepX mRNA-8 (Takara 640096) protocol. cDNA products were then amplified for 15 cycles of PCR using longAmp Taq (NEB M0287S). During amplification, PrepX RNAseq index barcode primers were added for each library to enable multiplexing. The amplified library was then cleaned up using PrepX PCR cleanup-8 protocol with magnetic beads (Aline C-1003). The final cDNA libraries were quantified using a high sensitivity dsDNA kit (Thermo Fisher Q32854) on a Qubit 3.0 Fluorometer (Thermo Fisher Q33216). cDNA content and quality were assessed with D1000 (Agilent 5067-5582) or High sensitivity D1000 tape (Agilent 5067-5584, when cDNA was in low amounts) on an Agilent Tapestation 2200 or 4200. For a step-by-step detailed protocol see Supplementary File S1.

### Sequencing cDNA libraries

Libraries were sequenced on an Illumina HiSeq 2500 sequencer. Single end-50bp reads were sequenced on a high-throughput flow cell. Libraries of varying concentrations were normalized to be equimolar, the concentrations of which ranged between 2 and 10 nM per lane. All the samples in a flow cell were multiplexed and later separated based on unique prepX indices to yield at least 10 million reads per library. The reads were demultiplexed and trimmed of adapters using the bcl2fastq2 v2.2 pipeline to yield final fastq data files.

### RNA-seq data processing

The *D. melanogaster* genome assembly and gene annotations were obtained from FlyBase version dmel_r6.36_FB2020_05 (Larkin *et al.* 2021). The gene expression in each library was quantified with RSEM v1.3.3 (Li and Dewey 2011) using STAR v2.7.6a as read aligner (Dobin *et al.* 2013). Because some of the tissue-specific biological samples were sequenced in more than one lane or run, and therefore the reads were split into multiple fastq files, the gene counts belonging to the same biological sample were summed. Gene counts in each dataset were normalized with the variance-stabilizing transformation (VST) method implemented in the DESeq2 v1.26.0 (Love *et al.* 2014) R package. Further analyses, such as principal component analysis (PCA), hierarchical clustering, and differential expression (DE) analysis, were performed in R using the VST-normalized counts.

### Differential Expression (DE) analysis

The DE analyses were performed with DESeq2 v1.26.0 (Love *et al.* 2014). On the whole ovary dataset, the contrasts tested were early *vs* mid, and mid *vs* late stages. For the tissue-specific datasets, three different comparisons were performed. First, to identify differentially expressed genes independently of the stage, all stages of somatic cells were compared to all stages of germ cells. Second, to identify genes up-regulated in a stage-specific manner within each tissue, we compared the expression level at each stage to the mean expression level of the other two stages. Third, we compared germ cells and somatic cells independently at each stage. Genes with a Benjamini-Hochberg (BH) adjusted *P*-value lower than 0.01 were selected as differentially expressed in the corresponding contrast.

### Functional analysis

The gene ontology (GO) and Kyoto Encyclopedia of Genes and Genomes (KEGG) pathways enrichment analyses were performed on the differentially expressed genes with the enrichGO and enrichKEGG functions of the clusterProfiler package (v3.14.3) for R (Yu *et al.* 2012). The GO terms were obtained using the R package AnnotationDbi (Carlson 2015) with the database org. Dm.eg.db v3.10.0. The GO overrepresentation analysis of biological process (BP) was performed against the gene universe of all *D. melanogaster* annotated genes in org. Dm.eg.db, adjusting the *P*-values with the BH method, adjusted *P*-value and *q*-value cutoff of 0.01, and a minimum of 30 genes per term. For the KEGG enrichment analysis, *P*-values were adjusted by the BH procedure, and an adjusted *P*-value cutoff of 0.05 was used.

## Results

### Staging larval ovary development during terminal filament formation

We divided the developing *Drosophila* larval ovary into three stages during terminal filament formation and used RNA-seq to quantify gene expression at these stages (Figure 1A). First, we considered an early stage of terminal filament formation at the early third instar larva (72 h After Egg Laying, 72 h AEL), when terminal filament assembly is initiating (Godt and Laski 1995) (Figure 1A-i). Second, we assigned the mid stage (96 h AEL) as 24 h after the early stage, at the midway point of terminal filament assembly (Godt and Laski 1995) (Figure 1A-ii). Third, the late stage (120 h AEL) was defined as the time point of white pupa formation (when the larvae become immobile at the larval to pupal transition; Ashburner *et al.* 2005), which occurs 24 h after the mid stage (Figure 1A-iii). At the white pupa stage, terminal filament assembly is complete and the number of terminal filaments reflects the number of adult ovarioles (Hodin and Riddiford 2000; Figure 1B).

**Figure 2:**
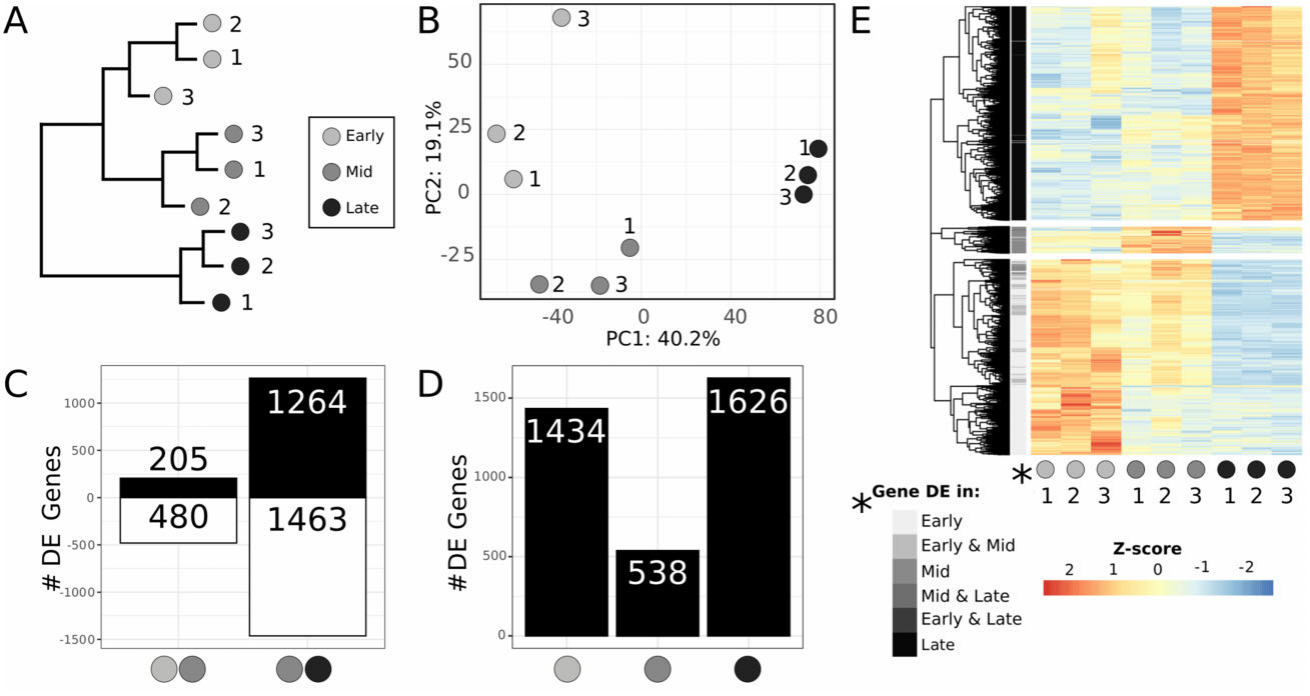
Whole ovary RNA-seq dataset overview. (A) hierarchical clustering dendrogram and (B) PCA of the whole ovary RNA-seq dataset, both showing that biological replicates are similar to each other, and that early and mid-stages are more similar to each other than either of them is to late stage. (C) Number of differentially expressed genes between early and mid stages, and between mid and late stages (adjusted *P*-value < 0.01; black: upregulated genes; white: downregulated genes). See Supplementary Table S2 for gene list. (D) Number of significantly upregulated stage-specific genes (adjusted *P*-value < 0.01). See Supplementary Table S3 for gene list. (E) Heatmap showing the expression of all the stage-specific upregulated genes as a row-wise z-score. Genes are clustered hierarchically and separated into three groups using the function “cutree,” and grayscale row labels (“*Gene DE”) immediately to the right of the tree are colored based on the stage in which the gene was detected to be significantly upregulated (*x*-axis categories).

We dissected these three stages of developing ovaries from larvae obtained from synchronized eggs and sequenced the transcripts present at each stage from pools of 30–100 ovaries (Supplementary Table S1). We aligned reads to the *D.melanogaster* genome (FlyBase v6.36), which yielded between 88.49% and 98.06% of reads aligned per sample (Supplementary Figure S1 and Table S1). Clustering analysis based on the VST of the gene counts of each sample confirmed that the three biological replicates of each stage clustered together, and that the three stages were well separated, as reflected by the dendrogram of the hierarchical analysis and the PCA (Figure 2, A and B). Furthermore, the dendrogram visualization of the hierarchical clustering results revealed that the mid stage was more similar in expression profile to the early stage than to the late stage. This indicates a more pronounced transcriptomic change at the transition from mid to late, than from early to mid, despite the fact that the same chronological amount of time had elapsed between each stage.

### Differential gene expression analysis of whole ovary samples at different stages

We analyzed the transcriptional differences between each stage and the successive one, thus performing a DE analysis comparing early to mid and mid to late transitions, using DESeq2 (Love *et al.* 2014) with a threshold of *P* < 0.01 (see *Materials andMethods*). We found a significantly higher number of genes differentially expressed in the mid to late transition (2727 genes), than in the early to mid-transition (685) (Figure 2C, Supplementary Table S2). Interestingly, from early to mid stages twice as many genes were downregulated (480) as upregulated (205), while from mid to late stages approximately the same number of genes were upregulated (1264) and downregulated (1463). We then identified the genes that were differentially expressed in one stage as compared to the other two stages, with the aim of revealing genes with stage-specific over- or under-expression. We found that early and late stages had many more over-expressed genes (1434 and 1626, respectively) than the mid stage (538) (Figure 2D, Supplementary Table S3). A heatmap representing the expression levels of the stage-specific overexpressed genes clearly separates the three groups of genes (Figure 2E). The first group in the heatmap contains the 1478 genes that are highly expressed specifically at early stages, with less expression at mid stages and very low expression at the late stage. Another large group of 1618 genes are highly expressed specifically at late stages and show low expression at early and mid stages. Finally, we identified a third and smallest group of 202 genes that are highly expressed at mid stages, with some detectable expression at early stages, but little detectable expression at the late stage (Figure 2E). These results are consistent with our previous observation that there is a high gene expression similarity in early and mid stages, and an increased transcriptomic change from mid to late stages.

### Separation of somatic and germ line tissues in the developing ovary

Given our ultimate interest in gene regulatory functions and dynamics during terminal filament formation, we wished to understand the predicted functions of the many differentially expressed genes across stages. We reasoned, however, that given the different developmental numbers, roles and behaviors of germ line and somatic cells in this developing organ, considering functional categories of differentially expressed genes in these whole ovary samples would be only minimally informative. We therefore designed an experimental strategy that allowed us to consider the transcriptional dynamics of the germ line and soma separately, described below.

To understand the gene expression differences between the somatic and germ line tissues of the ovary during terminal filament morphogenesis, we drove somatic and germ line tissue-specific GFP expression using the UAS-GAL4 system (Brand and Perrimon 1993), using the drivers *bab: GAL4* and *nos:GAL4*, respectively (see *Materials and Methods*). The paralogous genes *bab1* and *bab2* are expressed in somatic ovarian cells and are essential for terminal filament formation, differentiation of terminal filament cells, and stem cell niche maintenance (Sahut-Barnola *et al.* 1995; Couderc *et al.* 2002; Saler *et al.* 20120). The two Bab proteins act in a synergistic and partially redundant fashion in the ovary, and mutations in the *bab* genes cause defects in terminal filament and ovariole number (Godt and Laski 1995; Sahut-Barnola *et al.* 1995; Couderc *et al.* 2002). The gene *bab1* is expressed strongly in the apical and terminal filaments of the larval ovary, while *bab2* is expressed in swarm cells, in addition to other somatic cells of the larval ovary (Cabrera *et al.* 2002; Couderc *et al.* 2002). We used the *bab pGAL^4-2 ^*line, which was made by replacing a LacZ-carrying P-element insertion with a Gal4 element (P [Gal4, w+]) into the *bab^P^* site in the intronic region of *bab1* gene (Cabrera *et al.* 2002). The driver *bab: GAL4* (genotype: w[*]; P{bab1[Pgal^4-2^]/TM6B, Tb[1]) is expressed in all larval ovarian somatic cells (Cabrera *et al.* 2002; Couderc *et al.* 2002; Sarikaya *et al.* 2012; Saler *et al.* 2020). We used this *bab: GAL4* transgenic line to express GFP in larval ovarian somatic cells, allowing us to separate the somatic tissue from the germ line tissue using Fluorescence-activated cell sorting (FACS).

The expression of the gene *nanos* (*nos*) is limited to germ line cells in the larval ovary (Wang and Lin 2004). We used a *nos: Gal4* transgenic line (genotype: *P{w[+mC]=UAS-Dcr-2.D}1, w[1118]; P{w[+mC]=GAL4-nos.NGT}40*) to express GFP exclusively in the germ line cells, and thus isolate the germ line cells using FACS (Tracey *et al.* 2000).

We dissociated ovaries at the three stages described above and isolated the GFP-positive cells at each stage using FACS. Cellular debris was eliminated with gate R1, nonsinglets were eliminated by gate R2, and the R3 gate selected for GFP positive cells. A combination of the three gates yielded singlet GFP positive cells, minimizing the possibility of tissue contamination by undissociated cells. When similar numbers of ovaries were used to obtain sorted cells for somatic and germ line tissue-types, we found a larger number of somatic cells as compared to germ cells as expected, indicating a successful separation of the desired tissue type (Supplementary Figure S2).

**Figure 3:**
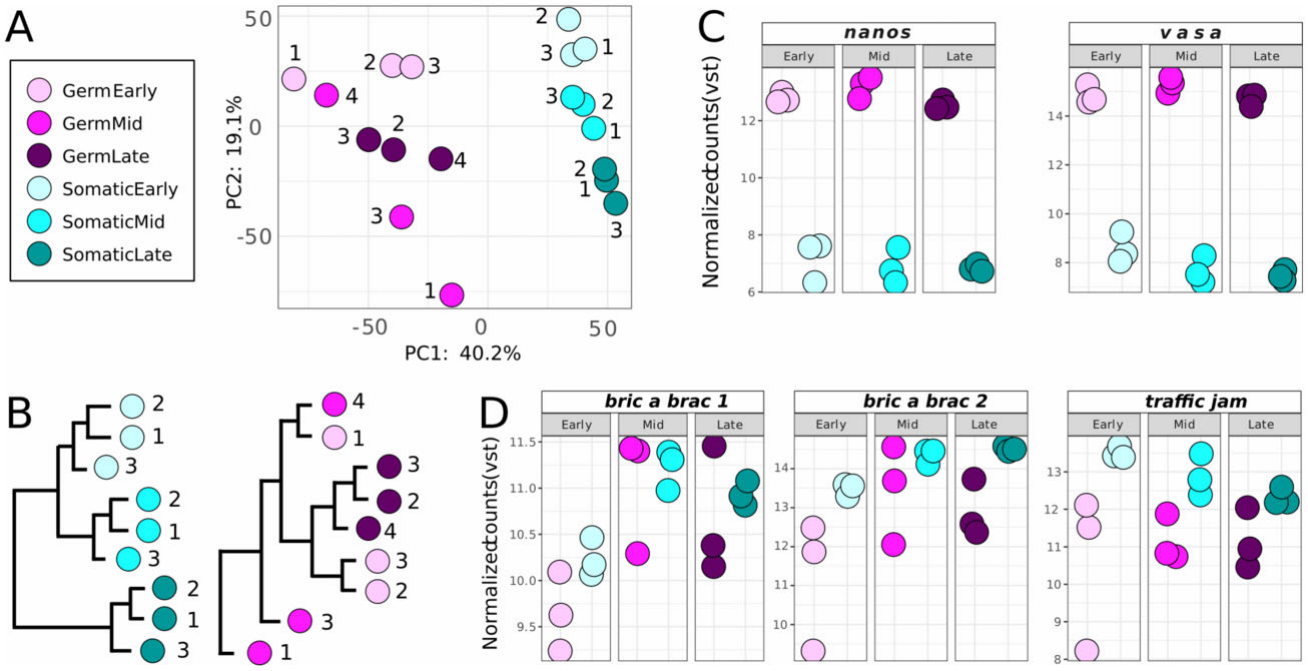
Cell type-specific RNA-seq dataset concordance and positive controls. (A) PCA Plot and (B) hierarchical clustering dendrogram of germ cell and somatic cell RNA-seq libraries. Expression in normalized counts by VST in each of the cell-type-specific RNA-seq libraries of (C) known germ cell markers *nanos* and *vasa*, and (D) known somatic cell markers *bric a brac 1*, *bric a brac 2*, and *traffic jam*.

With this method, we obtained tissue-specific transcriptomes of somatic and germ line tissues at the same three stages of terminal filament development used to generate the whole ovary dataset. We sequenced three biological replicates for all datasets and retained replicates that had at least 10 million reads. The number of reads aligned to the genome ranged from 11.0 to 81.5 million. Greater than 94.09% of reads aligned in all datasets, with the single exception of one dataset (Mid-1) with 88.55% of aligned reads (Supplementary Table S1 and Figure S3). The PCA analysis based on the counts normalized by VST shows a clear separation of somatic and germ cell libraries along the first principal component, suggesting a successful separation of cell types by FACS (Figure 3A). For the somatic samples, the three biological replicates cluster closely together (Figure 3, A and B) while the different stages are separated from each other in the second principal component. The structure of the dendrogram for the somatic samples resembles that of the whole ovary, in which early and mid stages are closer to each other than either is to the late stage. As for the germ cell libraries, unlike the biological replicates of the early and late stages, the mid stage replicates do not cluster together. A possible explanation is the low number of reads from the sample Mid-1 (Supplementary Table S1 and Figure S3).

To further assess the successful separation of somatic and germ cells, we checked the expression of well-known tissue-type-specific markers. The genes *nanos* and *vasa* are two genes known to be specifically expressed in germ cells in the ovary (Schupbach and Wieschaus 1986; Lehmann and Nusslein-Volhard 1991). Both genes show higher expression in the germ cell libraries than in the somatic cell libraries [mean log_2_(Fold Change) of 8.37 for *nanos*, and 8.23 for *vasa*] (Figure 3C), confirming that the preparation and sequencing of the germ cell libraries successfully captured the germ cells and their RNAs, and suggesting that germ cells were not present (or present only at very low levels) in the somatic cell libraries. *bab1*, *bab2*, and *tj* are considered ovarian somatic cell markers (Sahut-Barnola *et al.* 1995; Couderc *et al.* 2002). These three somatic markers display higher expression levels in our somatic libraries than in the germ cell libraries at each stage [mean log_2_(Fold Change) −0.31 for *bab1*, −1.31 for *bab2*, and −1.70 for *tj*] (Figure 3D). However, in four of the 18 libraries, either *bab1* or *bab2* (but not *tj*) showed higher expression levels in a specific germ cell library than in the somatic libraries. These specific cases were as follows: (1) one early stage germ cell replicate had higher *bab1* levels than one of the early somatic replicates; (2) two mid stage germ cell replicates had higher *bab1* levels than the somatic replicates; (3) one late stage germ cell replicate had higher *bab1* levels than the somatic replicates; (4) one mid stage germ cell replicate had higher *bab2* levels than the somatic replicates. This could indicate that some somatic cells might have been included in these particular germ cell libraries. Nonetheless, despite this putative small amount of contamination, we can clearly differentiate both tissue types based on their expression profiles as shown in the PCA (Figure 3A), suggesting that we captured the transcriptional differences between cell types (Figure 3A) sufficiently to allow us to achieve our goal of successfully retrieving the genes that are highly and differentially expressed in each of these two tissues.

###  Differential gene expression analysis of somatic and germ line tissues across all stages

**Figure 4:**
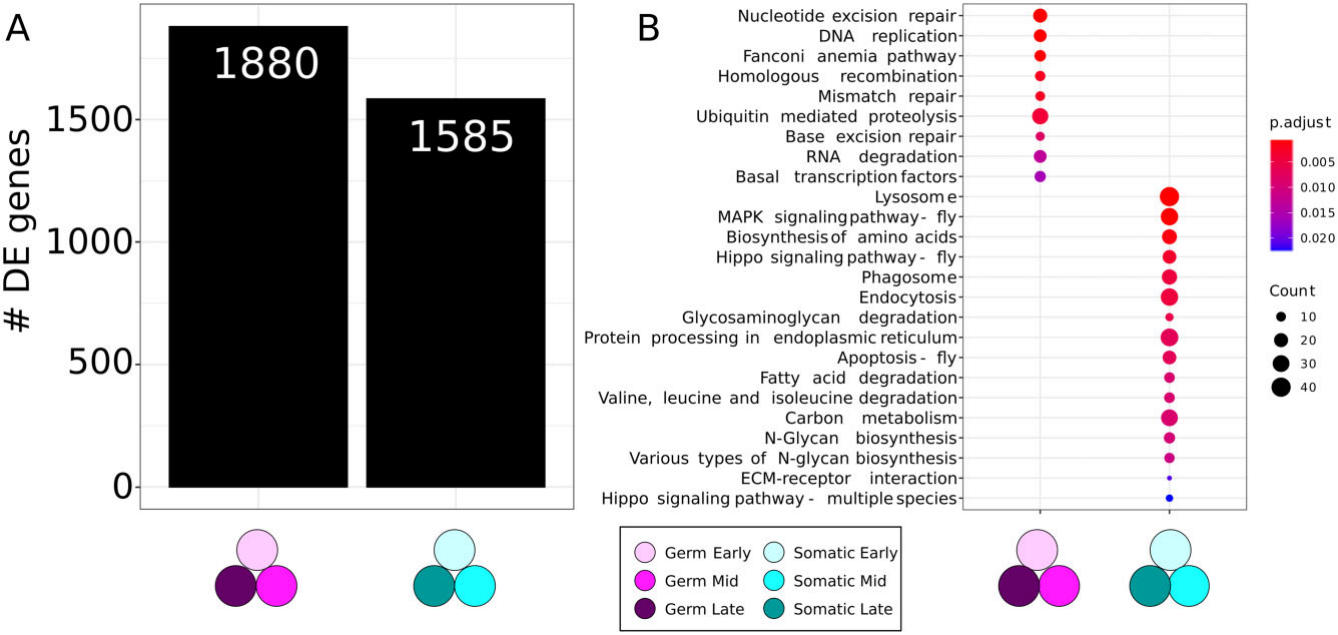
Transcriptomic differences between germ cells and somatic cells. (A) Number of significantly upregulated genes (adjusted *P*-value < 0.01) in germ cells and somatic cells. See Supplementary Table S4 for gene list. (B) Significantly enriched KEGG pathways (adjusted *P*-value < 0.05) within the upregulated genes of each cell type. The circle size is proportional to the number of differentially expressed genes that the indicated KEGG pathway contains, and the color gradient indicates the *P*-value.

The DE analysis between the somatic and germ line tissues across all three stages revealed 1880 genes significantly upregulated (adjusted *P*-value < 0.01) in germ cells and 1585 genes significantly upregulated in the somatic cells (Figure 4A; Supplementary Table S4).

Among the 20 most significant genes (with the lowest adjusted *P*-value) overexpressed in germ cells relative to somatic cells, we detected known germ line-specific genes including piRNA biogenesis genes *Argonaute3 (AGO3)*, *krimper (krimp)*, and *tejas (tej)*, along with *Aubergine (aub)*, (Brennecke *et al.* 2007; Olivieri *et al.* 2010; Patil and Kai 2010; Sato *et al.* 2015), *sisters unbound (sunn)* (Krishnan *et al.* 2014)*, benign gonial cell neoplasm (bgcn)* (Ohlstein *et al.* 2000), and uncharacterized genes including *CG32814* and *CG12851* on chromosome 2R. As for the somatic cells, the most significantly overexpressed gene relative to the germ cells is the cytochrome gene *Cyp4p2*, whose role is unknown in the ovary, followed by cytochrome *Cyp4p1* and the uncharacterized genes CG32581 and CG42329. Some genes known to play roles in the ovary were also among this group, including the regulator of the niche cells and ecdysone receptor *Taiman (tai)* (König *et al.* 2011), and the regulator of vitellogenesis *apterous (ap)* (Gavin and Williamson 1976).

### Temporally dynamic expression of genes previously studied in somatic ovary development

We explored the expression dynamics of some of the previously studied genes expressed in the *Drosophila* ovary. To our knowledge, temporal gene expression studies in the larval ovary for many of these genes have not yet been conducted.

First, we considered the temporal expression patterns of some adhesion proteins known to play a role in ovary development. *RanBPM* is an adhesion linker protein expressed in the germ line niche in the adult ovary (Dansereau and Lasko 2008). In our dataset, we see opposing trends of expression levels in somatic and germ line tissues, such that in germ line tissue *RanBPM* expression decreases progressively from early to mid to late stages, while in the somatic tissue it increases from early to mid to late stages (Supplementary Figure S4A). Cofilin (encoded by the gene *twinstar*) is an adhesion protein required for terminal filament cell rearrangement during terminal filament morphogenesis, as well as for adult border cell migration (Chen *et al.* 2001). Cofilin shows similar germ line and somatic cell expression trends, with higher levels at early stages that decrease progressively at mid and late stages (Supplementary Figure S4B).

We then looked at temporal expression of *RhoGEF64C* and *Wnt4*, genes involved in cell motility*.* RhoGEF64C is a small apically localized RhoGTPase that regulates cell shape and migration in the ovary (Simoes *et al.* 2006). In our datasets, we found *RhoGEF64C* expressed at higher levels in early and late stage somatic cells than at mid stages (Supplementary Figure S4C). *Wnt4* is involved in cell motility during ovarian morphogenesis (Cohen *et al.* 2002) and is expressed in the posterior terminal filaments and other somatic cell types of the third instar larval ovary (Slaidina *et al.* 2020). We found *Wnt4* to be expressed in lower levels in early and mid stages, with significantly increased expression in the late stage (Supplementary Figure S4D).

We also examined the temporal expression dynamics of a number of terminal filament cell-type-specific genes previously identified in a single cell sequencing study of the late third larval instar ovary (Slaidina *et al.* 2020). For example, *Diuretic hormone 44 receptor 2 (Dh442)* was identified as highly expressed in terminal filament cells (Slaidina *et al.* 2020). In our datasets, we observed a significant increase in expression levels only at the late stage relative to early and mid stage expression levels (Supplementary Figure S4E). Additional genes known to function in terminal filaments are *engrailed*, *invected*, *hedgehog*, and *patched* (Forbes *et al.* 1996; Besse *et al.* 2005; Bolívar *et al.* 2006; Saler *et al.* 2020). In our datasets, we observed *engrailed* and its paralog *invected* expressed at lowest levels at the early stage, showing a progressive increase in expression levels from mid to late stages (Supplementary Figure S4, F and G). Interestingly, *invected*, but not *engrailed*, showed significant differential expression between early and mid stages. The genes *patched* and *hedgehog* also showed significant increase from early to mid stage (Supplementary Figure S4, H and I).

Finally, we considered members of the fibroblast growth factor (FGF) signaling pathway, which controls sheath cell proliferation in the pupal ovary (Irizarry and Stathopoulos 2015). Three key genes of this pathway, the FGF ligand *thisbe*, the FGF scaffolding protein *stumps* and the upstream FGF signaling activator *heartless*, show significantly higher differential expression levels at early to mid stages than at mid to late stages (Supplementary Figure S4, J–L). These temporal profiles add to our understanding of the roles of these genes in ovarian morphogenesis by suggesting distinct putative critical regulatory periods for different genetic pathways.

### Functional enrichment analysis of differentially expressed genes in somatic and germ line tissues across all stages

To gain insight into the general functional categories of genes likely involved in ovarian germ cell and somatic behaviors during terminal filament development, we performed a GO enrichment analysis of the biological processes (BPs) of differentially expressed genes across cell types and developmental stages (Ashburner *et al.* 2000). We found 31 level four GO-terms enriched (adjusted *P*-value < 0.05) within the upregulated genes in germ cells, and 188 level four GO-terms enriched in the upregulated genes in somatic cells (Supplementary Figure S5). This analysis highlighted clear differences in the biological functions performed by the genes expressed in each tissue. The GO-terms enriched in the germ cells are primarily related to meiotic processes (9/31 contain the words “meiosis” or “meiotic”), chromosome stability (6/31 contain the words “chromosome” or “karyosome”) and cell cycle (12/31 contain “cell cycle”). In contrast, the GO-terms enriched in the somatic cells are principally related to cellular response (21/188 contain “response”), development (18/188), growth (16/188), morphogenesis (10/188), cell migration (6/188 contain the word “migration”), and signaling pathways (6/188).

To complement this GO enrichment analysis, we performed a KEGG pathway enrichment analysis on the same cell-type-specific overexpressed genes. The KEGG pathway database is a manually curated database of molecular interactions used to study enrichment of genetic regulatory pathways in gene lists (Kanehisa and Goto 2000). With this analysis, we identified nine KEGG pathways significantly enriched in the germ cells, and 16 significantly enriched pathways in the somatic cells (adjusted *P*-value < 0.05) (Figure 4B). The KEGG pathways enriched in the germ cells are generally related to meiosis and genome protection, while upregulated genes in the somatic cells are enriched for pathways involved in cell proliferation and cell death, including the previously identified *Hippo* (Barry and Camargo 2013; Sarikaya and Extavour 2015; Zheng and Pan 2019) and *MAPK* (Shaul and Seger 2007) signaling pathways.

### Stage- and tissue-specific differential gene expression analysis

To explore the functions of the stage-specific upregulated genes in each tissue type, we performed a DE analysis of somatic and germ line tissue at each of the three stages (Supplementary Figure S6 and Table S5) and then performed a GO analysis of biological functions and KEGG pathway enrichment analysis on the six sets of differentially expressed genes (upregulated at early, mid, and late stages in germ and somatic cells). The GO enrichment analysis of the genes differentially expressed in somatic cells over time (Supplementary Figure S6, A–C) revealed that four key BPs are consistent throughout all three stages including the mid stage, which has the smallest number of differentially expressed genes across stages. Specifically, these are the GO terms taxis, cell growth, actin filament-based process, and cell adhesion. At early and late stages, we additionally observe many key BPs related to morphogenesis in the somatic cells, including cell proliferation, differentiation, and migration.

**Figure 5:**
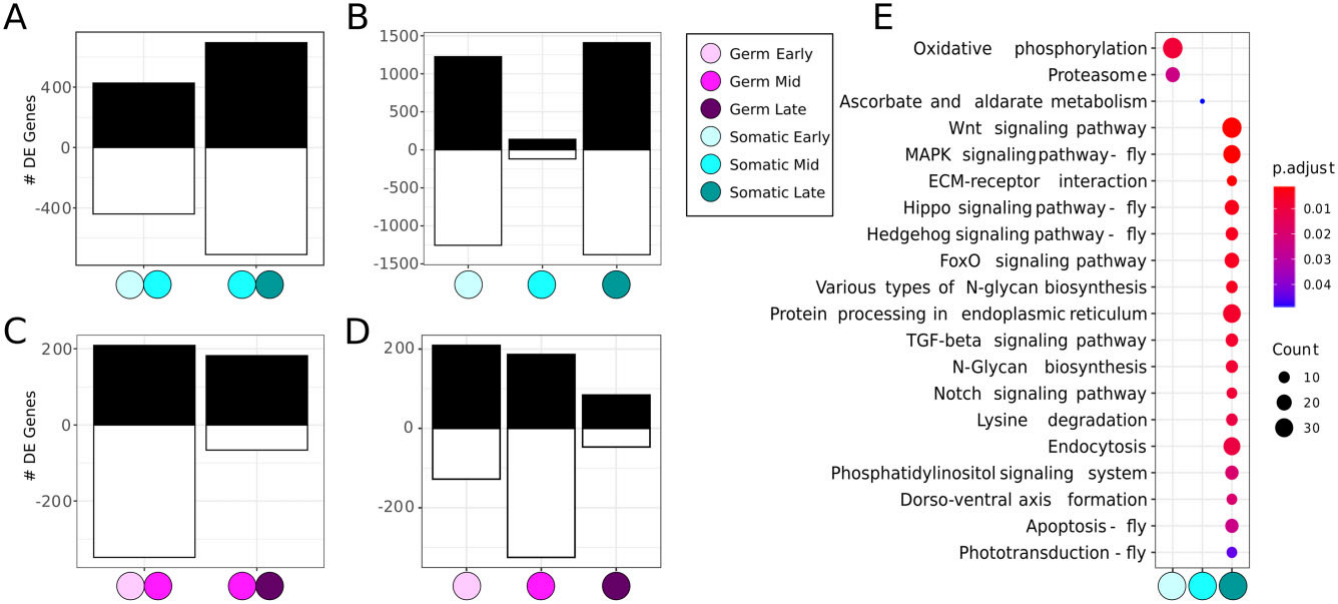
Cell type-specific DE analysis. (A) Number of differentially expressed genes (adjusted *P*-value < 0.01) upregulated (black) and downregulated (white) in somatic cells between early and mid, and between mid and late stages. See Supplementary Table S6 for gene list. (B) Number of differentially expressed genes (adjusted *P*-value < 0.01) upregulated (black) and downregulated (white) in somatic cells at each stage compared to the two other stages. See Supplementary Table S7 for gene list. (C) Number of differentially expressed genes (adjusted *P*-value < 0.01) upregulated (black) and downregulated (white) in germ cells between early and mid, and between mid and late stages. See Supplementary Table S8 for gene list. (D) Number of differentially expressed genes (adjusted *P*-value < 0.01) upregulated (black) and downregulated (white) in germ cells at each stage compared to the two other stages. See Supplementary Table S9 for gene list. (E) Significantly enriched (adjusted *P*-value < 0.05) KEGG pathways within the upregulated genes at each somatic stage. Circle size is proportional to the number of differentially expressed genes it contains, and the color gradient indicates the *P*-value.

To obtain a finer-grained view of the dynamic regulation of ovary development during terminal filament formation, we also performed DE analysis and functional enrichment analysis of somatic and germ line tissue types at each of the three stages. In the somatic cells, the number of differentially expressed genes between the early and mid stages (867 genes) is lower than between the mid and late stages (1404 genes) (Figure 5A; Supplementary Table S6). To identify genes with stage-specific upregulation, we compared each stage to the other two stages. We identified a higher number of stage-specific upregulated genes in early stages (1227) and late stages (1409) than at mid stages (139) (Figure 5B; Supplementary Table S7).

The germ cells, in general, display fewer differentially expressed genes between stages than the somatic cells. From early to mid stages there are twice as many differentially expressed genes (557 genes) as from mid to late stages (248 genes) (Figure 5C; Supplementary Table S8). In terms of stage-specific upregulated genes, the highest number of such genes is found at early stages (209), followed by mid (186), and late (84) stages (Figure 5D; Supplementary Table S9). The 1227, 139, and 1409 genes found upregulated at early, mid, and late stages, respectively of somatic tissue were enriched for two KEGG pathways at early stages, one at mid stage and 17 in late stages (Figure 5E). This analysis allowed us to pinpoint the stage(s) at which specific pathways were enriched in somatic cells relative to germ cells, which included apoptosis, Hippo signaling, and MAPK signaling. In addition, we detected some signaling pathways enriched only in somatic cells at late stages, such as the *Hedgehog*, *FoxO*, and *Notch* pathways (Figure 5E).

Given the known role of the Hippo pathway in cell proliferation (Wu *et al.* 2003; Huang *et al.* 2005; Barry and Camargo 2013), and specifically in terminal filament cell and terminal filament number regulation (Sarikaya and Extavour 2015), we proceeded to analyze the expression patterns of the genes belonging to the core Hippo signaling pathway. We found that most Hippo pathway core genes display increasing expression levels from early to mid to late stages, with the exception of the expression of the core gene *Rae1*, which progressively decreases in expression level from early to late stages (Supplementary Figure S7).

In the germ cells, across stages we find fewer processes directly involved in development and morphogenesis, with GO categories belonging to meiosis and cell cycle (Supplementary Figure S6). Among the 209, 186, and 84 upregulated genes in germ cells at early, mid, and late stages respectively, only one KEGG pathway (Ribosome) and one BP GO-term (cytoplasmic translation) were found significantly enriched at early stages. No such enrichment was detected at mid stages, and three KEGG pathways were enriched at late stages (Supplementary Figure S8).

### Uncharacterized genes

The detection of uncharacterized genes among the top differentially expressed genes in germ cells drove us to ask if there were any differences in the proportion of uncharacterized genes in each set of differentially expressed genes. We found that in the genes significantly upregulated in somatic cells compared to germ cells, 29.15% are categorized as “uncharacterized proteins” in FlyBase (Larkin *et al.* 2021), while within the significantly upregulated genes in germ cells, the proportion of uncharacterized genes was 39.10%. Within the stage-specific upregulated genes, the proportion of uncharacterized genes remained constant (between 28.96% and 29.63%) in somatic cells, while in germ cells it increased from 29.08% in early stages, to 34.83% in mid stages, and to 37.40% in late stages (Supplementary Figure S9).

### Expression of cell type-specific markers

A previous single cell RNA-sequencing dataset of the late third stage larval ovary (Slaidina *et al.* 2020) identified transcriptional profile clusters interpreted as indicative of cell types, and suggested gene markers associated with each cell type. To determine whether the cell types identified at this late stage might also be present at earlier developmental stages than those previously assessed, we examined the expression levels of those suggested markers across our datasets. As expected, the majority of the germ cell markers are highly expressed in our germ cell libraries and expressed only at low levels in the somatic cells (Supplementary Figure S10). Among the somatic markers detected in our somatic tissue libraries, we do not observe any particular temporal expression pattern specific to a given somatic cell type. Nevertheless, we clearly distinguish two groups of somatic markers (Supplementary Figure S11). One group is composed of somatic markers whose expression levels are highest at early and mid stages, and decay at the late stage, and a larger group of makers that are less strongly expressed at early stages, show increased expression at the mid stage, and show the highest expression at late stages. By contrast, the germ cell markers detected in our germ cell libraries do not display any clear temporal expression pattern. Instead, most of these genes were expressed at similar levels across the three studied stages (Supplementary Figure S12). This is consistent with our previous observations that the germ line dataset is not enriched for any signaling pathway directly implicated in development during these three time points (Supplementary Figure S6).

## Discussion

### Temporal gene expression during ovary morphogenesis

We systematically staged and sequenced entire larval ovaries to generate a gene expression dataset during terminal filament formation. We then separated somatic and germ line tissues during these stages and generated tissue-specific transcriptomes. While the development of the *Drosophila* ovary has been studied for the last several decades, and progress has been made in identifying the roles of some signaling pathways in its morphogenesis (Cohen *et al.* 2002; Besse *et al.* 2005; Gilboa and Lehmann 2006; Gancz *et al.* 2011; Gancz and Gilboa 2013; Matsuoka *et al.* 2013; Gilboa 2015; Irizarry and Stathopoulos 2015; Lengil *et al.* 2015; Mendes and Mirth 2016; Panchal *et al.* 2017) to our knowledge, there are no publicly available transcriptomes of larval ovaries of *Drosophila* across developmental time. Recent articles have reported single cell RNA-sequencing for *Drosophila* ovaries, focusing either on a single larval time point or on adult ovaries (Jevitt *et al.* 2020; Rust *et al.* 2020; Slaidina *et al.*^2020^, ^2021^). Our stage and tissue-type specific data thus represent a valuable complementary transcriptomic resource on the morphogenesis of the larval ovaries of *Drosophila*, a complex process that ultimately influences reproductive capacity.

### Differential gene expression across developmental stages of the larval ovary

Both the whole ovary datasets and the somatic tissue datasets show increased numbers of differentially expressed genes from the mid to late stage transition, and in the late stage of terminal filament formation in the larval ovary (Figures 2D and 5A). In contrast, germ cells show higher numbers of differentially expressed genes in the early-to-mid stage transition, and in the early stages. The similarity in differentially expressed gene numbers and signaling pathways in the whole ovary and somatic cell datasets suggests that because the somatic cells are higher in number than the germ cells (Supplementary Figure S2), their transcriptomes dominate the whole-ovary transcriptomes derived from late stages of larval ovary development. Further functional enrichment analyses of somatic and germ line tissue revealed that distinct functions and pathways likely operate in these two cell types during larval ovary development.

It is possible that germ cells may be especially sensitive to DNA damage given their role in propagating genetic material, which we speculate may explain the enrichment of processes related to nucleotide replication, recombination and repair in our analysis of the differentially expressed genes in germ cells (Figure 4B, Supplementary Figure S5). Similarly, we observed many genes of the piRNA pathway (*e.g.*, *AGO3*, *aub*, *krimp*, and *tej*), which protect the genome from transposable elements (Supplementary Table S5) (Sato and Siomi 2020) among the top significantly enriched genes in germ cells. On the other hand, the somatic tissue is enriched for different signaling pathways including Hippo, MAPK, and apoptosis (Figure 5E, Supplementary Figure S6), which are known to play a role in either larval or adult ovary morphogenesis (Lynch *et al.* 2010; Khammari *et al.* 2011; Elshaer and Piulachs 2015; Sarikaya and Extavour 2015). The observation of a higher number of uncharacterized genes in the germ line tissue datasets (Supplementary Figure S9) highlights the importance of future functional characterization of these genes to understand their possible roles in germ line gene regulation.

### Cell adhesion and migration during ovary morphogenesis

We assessed the temporal dynamics of genes expressed in specific cell types during development to serve as generators of new hypotheses to understand the role of genes and pathways during morphogenesis. *RhoGEF64C* is a RhoGTPase with some role in regulating control cell shape changes that lead to epithelial cell invagination (Simoes *et al.* 2006; Toret and Le Bivic 2021). In a genome-wide association study on ovariole number phenotypes in natural populations of *Drosophila*, *RhoGEF64C* driven in somatic tissue had a significant effect on adult ovariole number (Lobell *et al.* 2017). The significant increase in expression of *RhoGEF64C* we observed in early and late stages (Supplementary Figure S4C) suggests its role in somatic cell shape and migration in both early and late stages.

GO-terms related to cell adhesion, motility and taxis were enriched in all three stages in somatic cells (Supplementary Figure S6). Previous studies have shown signaling pathways involved in ovary development to affect cell adhesion and migration processes (Cohen *et al.* 2002; Li *et al.* 2003; Besse *et al.* 2005; Lai *et al.* 2017). Migratory events in mid to late stages of the larval ovary have been described for two ovarian cell types, swarm cells and sheath cells (Sahut-Barnola *et al.*^1995^, ^1996^; Green and Extavour 2012; Slaidina *et al.* 2020). The increased number of differentially expressed genes that correspond to the processes of cell migration and adhesion may be due to the migratory events in the mid to late stages of larval ovary morphogenesis.

The FGF signaling pathway supports terminal filament cell differentiation in the early larval stages through the ligand *thisbe (ths)* and the upstream activator *heartless (htl)*, and also controls sheath cell proliferation in late larval and pupal stages (Irizarry and Stathopoulos 2015). In our dataset, we observe that in somatic cells these FGF pathway genes show a significant progressive upregulation from early to mid and from mid to late stages (Supplementary Figure S4, J–L). Consistently, *ths* and *stumps* were identified as markers of a distinct migratory ovarian cell population, the sheath cells (Slaidina *et al.* 2020). The gene *stumps* is expressed in sheath cells in stages corresponding to our “late” stage in the differentiating terminal filament cells and in pupal stages (144 h AEL), of migratory sheath cells (Irizarry and Stathopoulos 2015).

### Functional enrichment analysis and signaling pathways

Our results show that in the late stage of somatic cells there is an increase in expression of genes involved in multiple signaling pathways, including the Wnt, MAPK, Hippo, Hedgehog, FoxO, TGF, and Notch pathways (Figure 5E). The molecular mechanisms of all these signaling pathways during larval ovary development have not yet been extensively studied, but all of them have been functionally implicated in ovariole number determination by a large-scale genetic screen (Kumar *et al.* 2020).

We previously showed that the Hippo signaling pathway controls proliferation of somatic cells, which affects terminal filament number (Sarikaya and Extavour 2015). Our differential gene expression data show that members of the Hippo pathway are significantly differentially expressed in the somatic tissue (Figure 4B; Supplementary Figure S8). Loss of function mutations in Yki, an effector of the Hippo signaling pathway, cause increased growth and reduced apoptosis through an increase in the levels of the cell cycle protein Cyc E and the apoptosis inhibitor Diap1 (Harvey *et al.* 2003; Huang *et al.* 2005). In our somatic cell datasets, we observe *Diap1* transcript levels significantly increase from early to late stages, and those of *CycE* increase from mid to late stages (Supplementary Figure S13, A and B). However, the apoptosis KEGG pathway appears significantly enriched in the somatic late stage (Figure 5E; Supplementary Figure S6). Furthermore, apoptosis-related genes *Dronc* and *Dark*, which form the apoptosome (Supplementary Figure S13, C and D) (Yuan *et al.* 2011), are also significantly upregulated in the late stage, as are the caspases *Dcp-1*, *Drice*, and *Dredd* (Supplementary Figure 13, C, E–G) (Harvey *et al.* 2001). Thus, we observe both an upregulation of apoptosis and an upregulation of the apoptosis inhibition genes in late stage somatic cells. This could mean that genes controlling apoptosis both positively and negatively are acting to exert tight control of this process. Alternatively, our observations may reflect that each process is upregulated within different somatic cell types.

Cap cells and intermingled cells are somatic cells that interact with the germ cells for the maintenance of germ line stem cell niches (Li *et al.* 2003; Song *et al.* 2007). The Notch signaling pathway, enriched in the late stage somatic dataset (Figure 5E; Supplementary Figure S6C), is required for cap cell fate (Panchal *et al.* 2017; Yatsenko and Shcherbata 2021). We observed an expression level increase in Notch pathway components at late stages, suggesting that the role of the Notch pathway in cap cell fate determination may be particularly important at mid to late stages of larval ovary development.

Components of the TGFβ pathway, enriched in late stage somatic cells in our dataset (Figure 5C), are known to contribute to ovarian development. These include the bone morphogenetic protein (BMP) and Activin pathways of the TGFβ pathway superfamily (Pangas and Woodruff 2000; Guo and Wang 2009). The BMP ligand *decapentaplegic* (*dpp)* was previously documented as expressed in all larval ovarian somatic cells and in cap cells of the late third instar larval ovary (Xie and Spradling 1998; Sato *et al.* 2010; Saler *et al.* 2020). The expression of *dpp* in the larval ovary is dependent on the expression of *bab* genes (Saler *et al.* 2020). The activin pathway controls terminal filament cell proliferation and differentiation (Lengil *et al.* 2015). We find that the activin receptor *baboon* shows a significant expression level increase in the late stage somatic cells (adjusted *P*-value of 0.002602), which could indicate its role in terminal filament cell differentiation in late stages.

## Conclusions

Here, we provide a dataset that explores gene expression during larval ovary development and morphogenesis, which is crucial to understand how the ovary is shaped in early stages to develop into a functional adult organ. This work offers a dataset for the developmental biology community to probe the genetic regulation of larval ovarian morphogenesis.

## Supplementary Material

jkab305_Table_S1

jkab305_Table_S2

jkab305_Table_S3

jkab305_Table_S4

jkab305_Table_S5

jkab305_Table_S6

jkab305_Table_S7

jkab305_Table_S8

jkab305_Table_S9

jkab305_Supplementary_File1

## Data Availability

All the raw data are publicly available at NCBI-Gene Expression Omnibus (GEO) database under the accession code GSE172015. The scripts used to process and analyze the data are available at GitHub repository https://github.com/guillemylla/Ovariole_morphogenesis_RNAseq. Supplementary material is available at *G3* online.

## References

[jkab305-B1] Ashburner M , Ball CA, Blake JA, Botstein D, Butler H, et al 2000. Gene ontology: tool for the unification of biology. The gene ontology consortium. Nat Genet. 25:25–29. 10802651 10.1038/75556 PMC3037419

[jkab305-B2] Ashburner M , Golic KG, Hawley RS. 2005. Drosophila: A Laboratory Handbook. Cold Spring Harbor, NY: Cold Spring Harbor Laboratory Press.

[jkab305-B3] Barolo S , Carver LA, Posakony JW. 2000. GFP and beta-galactosidase transformation vectors for promoter/enhancer analysis in Drosophila. Biotechniques. 29:726, 728, 730, 732. 10.2144/00294bm10 11056799

[jkab305-B4] Barry ER , Camargo FD. 2013. The Hippo superhighway: signaling crossroads converging on the Hippo/Yap pathway in stem cells and development. Curr Opin Cell Biol. 25:247–253. 23312716 10.1016/j.ceb.2012.12.006

[jkab305-B5] Besse F , Busson D, Pret AM. 2005. Hedgehog signaling controls Soma-Germen interactions during Drosophila ovarian morphogenesis. Dev Dyn. 234:422–431. 16145667 10.1002/dvdy.20537

[jkab305-B6] Bolivar J , Pearson J, Lopez-Onieva L, Gonzalez-Reyes A. 2006. Genetic dissection of a stem cell niche: the case of the Drosophila ovary. Dev Dyn. 235:2969–2979. 17013875 10.1002/dvdy.20967

[jkab305-B7] Brand AH , Perrimon N. 1993. Targeted gene expression as a means of altering cell fates and generating dominant phenotypes. Development. 118:401–415. 8223268 10.1242/dev.118.2.401

[jkab305-B8] Brennecke J , Aravin AA, Stark A, Dus M, Kellis M, et al 2007. Discrete small RNA-generating loci as master regulators of transposon activity in *Drosophila*. Cell. 128:1089–1103. 17346786 10.1016/j.cell.2007.01.043

[jkab305-B9] Büning J. 1994. The Insect Ovary: ultrastructure, Previtellogenic Growth and Evolution. London: Chapman and Hall.

[jkab305-B10] Cabrera GR , Godt D, Fang PY, Couderc JL, Laski FA. 2002. Expression pattern of Gal4 enhancer trap insertions into the bric a brac locus generated by P element replacement. Genesis. 34:62–65. 12324949 10.1002/gene.10115

[jkab305-B11] Carlson M. 2015. AnnotationDbi: Introduction to Bioconductor Annotation Packages. http://bioconductor.statistik.tu-dortmund.de/packages/2.11/bioc/vignettes/AnnotationDbi/inst/doc/IntroToAnnotationPackages.pdf.

[jkab305-B12] Chen J , Godt D, Gunsalus K, Kiss I, Goldberg M, et al 2001. Cofilin/ADF is required for cell motility during Drosophila ovary development and oogenesis. Nat Cell Biol. 3:204–209. 11175754 10.1038/35055120

[jkab305-B13] Church SH , de Medeiros BAS, Donoughe S, Marquez Reyes NL, Extavour CG. 2021. Repeated loss of variation in insect ovary morphology highlights the role of developmental constraint in life-history evolution. Proc R Soc Lond B Biol Sci. 288:20210150. 10.1098/rspb.2021.0150 PMC8097220 33947234

[jkab305-B14] Cohen ED , Mariol MC, Wallace RM, Weyers J, Kamberov YG, et al 2002. DWnt4 regulates cell movement and focal adhesion kinase during Drosophila ovarian morphogenesis. Dev Cell. 2:437–448. 11970894 10.1016/s1534-5807(02)00142-9

[jkab305-B15] Couderc JL , Godt D, Zollman S, Chen J, Li M, et al 2002. The bric a brac locus consists of two paralogous genes encoding BTB/POZ domain proteins and acts as a homeotic and morphogenetic regulator of imaginal development in Drosophila. Development. 129:2419–2433. 11973274 10.1242/dev.129.10.2419

[jkab305-B16] Dansereau DA , Lasko P. 2008. RanBPM regulates cell shape, arrangement, and capacity of the female germline stem cell niche in *Drosophila melanogaster*. J Cell Biol. 182:963–977. 18762575 10.1083/jcb.200711046 PMC2528568

[jkab305-B17] David J. 1970. Le nombre d'ovarioles chez la Drosophila: relation avec la fecondite et valeur adaptive [Number of ovarioles in *Drosophila melanogaster*: relationship with fertility and adaptive value. Arch Zool Exp Gen. 111:357–370.

[jkab305-B18] Dobin A , Davis CA, Schlesinger F, Drenkow J, Zaleski C, et al 2013. STAR: ultrafast universal RNA-seq aligner. Bioinformatics. 29:15–21. 23104886 10.1093/bioinformatics/bts635 PMC3530905

[jkab305-B19] Elshaer N , Piulachs MD. 2015. Crosstalk of EGFR signalling with Notch and Hippo pathways to regulate cell specification, migration and proliferation in cockroach panoistic ovaries. Biol Cell. 107:273–285. 25907767 10.1111/boc.201500003

[jkab305-B20] Forbes AJ , Lin H, Ingham PW, Spradling AC. 1996. hedgehog is required for the proliferation and specification of ovarian somatic cells prior to egg chamber formation in Drosophila. Development. 122:1125–1135. 8620839 10.1242/dev.122.4.1125

[jkab305-B21] Gancz D , Gilboa L. 2013. Insulin and Target of rapamycin signaling orchestrate the development of ovarian niche-stem cell units in Drosophila. Development. 140:4145–4154. 24026119 10.1242/dev.093773

[jkab305-B22] Gancz D , Lengil T, Gilboa L. 2011. Coordinated regulation of niche and stem cell precursors by hormonal signaling. PLoS Biol. 9:e1001202. 22131903 10.1371/journal.pbio.1001202 PMC3222635

[jkab305-B23] Gavin JA , Williamson JH. 1976. Juvenile hormone-induced vitellogenesis in Apterous4, a non-vitellogenic mutant in *Drosophila melanogaster*. J Insect Physiol. 22:1737–1742. 827589 10.1016/0022-1910(76)90067-6

[jkab305-B24] Gilboa L. 2015. Organizing stem cell units in the *Drosophila* ovary. Curr Opin Genet Dev. 32:31–36. 25703842 10.1016/j.gde.2015.01.005

[jkab305-B26] Gilboa L , Lehmann R. 2006. Soma-germline interactions coordinate homeostasis and growth in the Drosophila gonad. Nature. 443:97–100. 16936717 10.1038/nature05068

[jkab305-B27] Godt D , Laski FA. 1995. Mechanisms of cell rearrangement and cell recruitment in *Drosophila* ovary morphogenesis and the requirement of *bric à brac*. Development. 121:173–187. 7867498 10.1242/dev.121.1.173

[jkab305-B28] Green DA II , Extavour CG. 2012. Convergent evolution of a reproductive trait through distinct developmental mechanisms in *Drosophila*. Dev Biol. 372:120–130. 23022298 10.1016/j.ydbio.2012.09.014

[jkab305-B29] Green DA II , Extavour CG. 2014. Insulin signalling underlies both plasticity and divergence of a reproductive trait in Drosophila. Proc Biol Sci. 281:20132673. 24500165 10.1098/rspb.2013.2673 PMC3924071

[jkab305-B30] Guo X , Wang XF. 2009. Signaling cross-talk between TGF-beta/BMP and other pathways. Cell Res. 19:71–88. 19002158 10.1038/cr.2008.302 PMC3606489

[jkab305-B31] Harvey KF , Pfleger CM, Hariharan IK. 2003. The Drosophila Mst ortholog, hippo, restricts growth and cell proliferation and promotes apoptosis. Cell. 114:457–467. 12941274 10.1016/s0092-8674(03)00557-9

[jkab305-B32] Harvey NL , Daish T, Mills K, Dorstyn L, Quinn LM, et al 2001. Characterization of the Drosophila caspase, DAMM. J Biol Chem. 276:25342–25350., 11337486 10.1074/jbc.M009444200

[jkab305-B33] Hodin J. 2009. Chapter 11: She Shapes Events As They Come: Plasticity in Female Insect Reproduction. In: Whitman D, Ananthakrishnan TN, editors. Phenotypic Plasticity of Insects. Enfield, NH: Science Publishers, Inc. p. 423–521.

[jkab305-B34] Hodin J , Riddiford LM. 2000. Different mechanisms underlie phenotypic plasticity and interspecific variation for a reproductive character in drosophilids (Insecta: Diptera). Evolution. 54:1638–1653. 11108591 10.1111/j.0014-3820.2000.tb00708.x

[jkab305-B35] Honěk A , Honek A. 1993. Intraspecific variation in body size and fecundity in insects - a general relationship. Oikos. 66:483–492.

[jkab305-B36] Huang J , Wu S, Barrera J, Matthews K, Pan D. 2005. The Hippo signaling pathway coordinately regulates cell proliferation and apoptosis by inactivating Yorkie, the Drosophila Homolog of YAP. Cell. 122:421–434. 16096061 10.1016/j.cell.2005.06.007

[jkab305-B37] Irizarry J , Stathopoulos A. 2015. FGF signaling supports Drosophila fertility by regulating development of ovarian muscle tissues. Dev Biol. 404:1–13. 25958090 10.1016/j.ydbio.2015.04.023 PMC4469552

[jkab305-B38] Jevitt A , Chatterjee D, Xie G, Wang X-F, Otwell T, et al 2020. A single-cell atlas of adult Drosophila ovary identifies transcriptional programs and somatic cell lineage regulating oogenesis. PLoS Biol. 18:e3000538. 32339165 10.1371/journal.pbio.3000538 PMC7205450

[jkab305-B39] Kanehisa M , Goto S. 2000. KEGG: Kyoto Encyclopedia of Genes and Genomes. Nucleic Acids Res. 28:27–30. 10592173 10.1093/nar/28.1.27 PMC102409

[jkab305-B40] Khammari A , Agnes F, Gandille P, Pret AM. 2011. Physiological apoptosis of polar cells during Drosophila oogenesis is mediated by Hid-dependent regulation of Diap1. Cell Death Differ. 18:793–805. 21113144 10.1038/cdd.2010.141 PMC3131922

[jkab305-B41] King RC. 1970. Ovarian Development in Drosophila melanogaster. Robert C King, editor. New York: Academic Press. p. 227.

[jkab305-B42] King RC , Aggarwal SK, Aggarwal U. 1968. The development of the female *Drosophila* reproductive system. J Morphol. 124:143–166. 5654408 10.1002/jmor.1051240203

[jkab305-B43] König A , Yatsenko AS, Weiss M, Shcherbata HR. 2011. Ecdysteroids affect Drosophila ovarian stem cell niche formation and early germline differentiation. EMBO J. 30:1549–1562. 21423150 10.1038/emboj.2011.73 PMC3102283

[jkab305-B44] Krishnan B , Thomas SE, Yan R, Yamada H, Zhulin IB, et al 2014. Sisters unbound is required for meiotic centromeric cohesion in *Drosophila melanogaster*. Genetics. 198:947–965. 25194162 10.1534/genetics.114.166009 PMC4224182

[jkab305-B45] Kumar T , Blondel L, Extavour CG. 2020. Topology-driven analysis of protein-protein interaction networks detects functional genetic sub-networks regulating reproductive capacity. eLife. 9:e54082. 32901612 10.7554/eLife.54082 PMC7550192

[jkab305-B46] Lai CM , Lin KY, Kao SH, Chen YN, Huang F, et al 2017. Hedgehog signaling establishes precursors for germline stem cell niches by regulating cell adhesion. J Cell Biol. 216:1439–1453. 28363970 10.1083/jcb.201610063 PMC5412570

[jkab305-B47] Larkin A , Marygold SJ, Antonazzo G, Attrill H, Dos Santos G, et al; FlyBase Consortium. 2021. FlyBase: updates to the *Drosophila melanogaster* knowledge base. Nucleic Acids Res. 49:D899–D907. 33219682 10.1093/nar/gkaa1026 PMC7779046

[jkab305-B49] Lehmann R , Nusslein-Volhard C. 1991. The maternal gene nanos has a central role in posterior pattern formation of the Drosophila embryo. Development. 112:679–691. 1935684 10.1242/dev.112.3.679

[jkab305-B50] Lengil T , Gancz D, Gilboa L. 2015. Activin signaling balances proliferation and differentiation of ovarian niche precursors and enables adjustment of niche numbers. Development. 142:883–892. 25633355 10.1242/dev.113902

[jkab305-B51] Li B , Dewey CN. 2011. RSEM: accurate transcript quantification from RNA-Seq data with or without a reference genome. BMC Bioinformatics. 12:323.in 21816040 10.1186/1471-2105-12-323 PMC3163565

[jkab305-B52] Li M , Hu X, Zhang S, Ho MS, Wu G, et al 2019. Traffic jam regulates the function of the ovarian germline stem cell progeny differentiation niche during pre-adult stage in Drosophila. Sci Rep. 9:10124. 31300663 10.1038/s41598-019-45317-6 PMC6626045

[jkab305-B53] Li MA , Alls JD, Avancini RM, Koo K, Godt D. 2003. The large Maf factor Traffic Jam controls gonad morphogenesis in *Drosophila*. Nat Cell Biol. 5:994–1000. 14578908 10.1038/ncb1058

[jkab305-B54] Lobell AS , Kaspari RR, Serrano Negron YL, Harbison ST. 2017. The genetic architecture of ovariole number in *Drosophila melanogaster*: Genes with major, quantitative, and pleiotropic effects. G3 (Bethesda). 7:2391–2403. 28550012 10.1534/g3.117.042390 PMC5499145

[jkab305-B55] Love MI , Huber W, Anders S. 2014. Moderated estimation of fold change and dispersion for RNA-seq data with DESeq2. Genome Biol. 15:550. 25516281 10.1186/s13059-014-0550-8 PMC4302049

[jkab305-B56] Lynch JA , Peel AD, Drechsler A, Averof M, Roth S. 2010. EGF signaling and the origin of axial polarity among the insects. Curr Biol. 20:1042–1047. 20471269 10.1016/j.cub.2010.04.023 PMC2902724

[jkab305-B57] Markow TA , O'Grady PM. 2007. *Drosophila* biology in the genomic age. Genetics. 177:1269–1276. 18039866 10.1534/genetics.107.074112 PMC2147954

[jkab305-B58] Matsuoka S , Hiromi Y, Asaoka M. 2013. Egfr signaling controls the size of the stem cell precursor pool in the Drosophila ovary. Mech Dev. 130:241–253. 23376160 10.1016/j.mod.2013.01.002

[jkab305-B59] Mendes CC , Mirth CK. 2016. Stage-specific plasticity in ovary size is regulated by insulin/insulin-like growth factor and ecdysone signaling in Drosophila. Genetics. 202:703–719. 26715667 10.1534/genetics.115.179960 PMC4788244

[jkab305-B60] Ohlstein B , Lavoie CA, Vef O, Gateff E, McKearin DM. 2000. The Drosophila cystoblast differentiation factor, benign gonial cell neoplasm, is related to DExH-box proteins and interacts genetically with bag-of-marbles. Genetics. 155:1809–1819. 10924476 10.1093/genetics/155.4.1809 PMC1461197

[jkab305-B61] Olivieri D , Sykora MM, Sachidanandam R, Mechtler K, Brennecke J. 2010. An *in vivo* RNAi assay identifies major genetic and cellular requirements for primary piRNA biogenesis in Drosophila. EMBO J. 29:3301–3317. 20818334 10.1038/emboj.2010.212 PMC2957214

[jkab305-B62] Panchal T , Chen X, Alchits E, Oh Y, Poon J, et al 2017. Specification and spatial arrangement of cells in the germline stem cell niche of the Drosophila ovary depend on the Maf transcription factor Traffic jam. PLoS Genet. 13:e1006790. 28542174 10.1371/journal.pgen.1006790 PMC5459507

[jkab305-B63] Pangas SA , Woodruff TK. 2000. Activin signal transduction pathways. Trends Endocrinol Metab. 11:309–314. 10996525 10.1016/s1043-2760(00)00294-0

[jkab305-B64] Patil VS , Kai T. 2010. Repression of retroelements in Drosophila germline via piRNA pathway by the Tudor domain protein tejas. Curr Biol. 20:724–730. 20362446 10.1016/j.cub.2010.02.046

[jkab305-B65] Rust K , Byrnes LE, Yu KS, Park JS, Sneddon JB, et al 2020. A single-cell atlas and lineage analysis of the adult Drosophila ovary. Nat Commun. 11:5628. 33159074 10.1038/s41467-020-19361-0 PMC7648648

[jkab305-B66] Sahut-Barnola I , Dastugue B, Couderc J-L. 1996. Terminal filament cell organization in the larval ovary of *Drosophila melanogaster*: ultrastructural observations and pattern of divisions. Rouxs Arch Dev Biol. 205:356–363. 28306086 10.1007/BF00377215

[jkab305-B67] Sahut-Barnola I , Godt D, Laski FA, Couderc J-L. 1995. *Drosophila* ovary morphogenesis: analysis of terminal filament formation and identification of a gene required for this process. Dev Biol. 170:127–135. 7601303 10.1006/dbio.1995.1201

[jkab305-B68] Saler LMM , Hauser V, Bartoletti M, Mallart C, Malartre M, et al 2020. The bric-à-brac BTB/POZ transcription factors are necessary in niche cells for germline stem cells establishment and homeostasis through control of BMP/DPP signaling in the *Drosophila melanogaster* ovary. PLoS Genet. 16:e1009128. 33151937 10.1371/journal.pgen.1009128 PMC7643948

[jkab305-B69] Sarikaya DP , Belay AA, Ahuja A, Dorta A, Green DA, et al 2012. The roles of cell size and cell number in determining ovariole number in Drosophila. Dev Biol. 363:279–289. 22200592 10.1016/j.ydbio.2011.12.017

[jkab305-B70] Sarikaya DP , Extavour CG. 2015. The Hippo pathway regulates homeostatic growth of stem cell niche precursors in the *Drosophila* ovary. PLoS Genet. 11:e1004962. 25643260 10.1371/journal.pgen.1004962 PMC4333732

[jkab305-B71] Sato K , Siomi MC. 2020. The piRNA pathway in Drosophila ovarian germ and somatic cells. Proc Jpn Acad Ser B Phys Biol Sci. 96:32–42. 10.2183/pjab.96.003 PMC6974405 31932527

[jkab305-B72] Sato K , Iwasaki YW, Shibuya A, Carninci P, Tsuchizawa Y, et al 2015. Krimper enforces an antisense Bias on piRNA pools by binding AGO3 in the Drosophila germline. Mol Cell. 59:553–563. 26212455 10.1016/j.molcel.2015.06.024

[jkab305-B73] Sato T , Ogata J, Niki Y. 2010. BMP and Hh signaling affects primordial germ cell division in Drosophila. Zool Sci. 27:804–810. 10.2108/zsj.27.804 20887178

[jkab305-B74] Schupbach T , Wieschaus E. 1986. Maternal-effect mutations altering the anterior-posterior pattern of the Drosophila embryo. Rouxs Arch Dev Biol. 195:302–317. 28306055 10.1007/BF00376063

[jkab305-B75] Shaul YD , Seger R. 2007. The MEK/ERK cascade: from signaling specificity to diverse functions. Biochim Biophys Acta. 1773:1213–1226. 17112607 10.1016/j.bbamcr.2006.10.005

[jkab305-B76] Simoes S , Denholm B, Azevedo D, Sotillos S, Martin P, et al 2006. Compartmentalisation of Rho regulators directs cell invagination during tissue morphogenesis. Development. 133:4257–4267. 17021037 10.1242/dev.02588

[jkab305-B77] Slaidina M , Banisch TU, Gupta S, Lehmann R. 2020. A single-cell atlas of the developing Drosophila ovary identifies follicle stem cell progenitors. Genes Dev. 34:239–249. 31919193 10.1101/gad.330464.119 PMC7000915

[jkab305-B78] Slaidina M , Gupta S, Banisch T, Lehmann R. 2021. A single cell atlas reveals unanticipated cell type complexity in Drosophila ovaries. bioRxiv: 427703. 10.1101/gr.274340.120 PMC8494228 34389661

[jkab305-B79] Song X , Call GB, Kirilly D, Xie T. 2007. Notch signaling controls germline stem cell niche formation in the Drosophila ovary. Development. 134:1071–1080. 17287246 10.1242/dev.003392

[jkab305-B80] Toret CP , Le Bivic AL. 2021. A potential Rho GEF and Rac GAP for coupled Rac and Rho cycles during mesenchymal-to-epithelial-like transitions. Small GTPases. 12:13–19. 30032715 10.1080/21541248.2018.1502592 PMC7781753

[jkab305-B81] Tracey WD Jr , Ning X, Klingler M, Kramer SG, Gergen JP. 2000. Quantitative analysis of gene function in the Drosophila embryo. Genetics. 154:273–284. 10628987 10.1093/genetics/154.1.273 PMC1460918

[jkab305-B82] Twombly V , Blackman RK, Jin H, Graff JM, Padgett RW, et al 1996. The TGF-beta signaling pathway is essential for Drosophila oogenesis. Development. 122:1555–1565. 8625842 10.1242/dev.122.5.1555

[jkab305-B83] Wang Z , Lin H. 2004. Nanos maintains germline stem cell self-renewal by preventing differentiation. Science. 303:2016–2019. 14976263 10.1126/science.1093983

[jkab305-B84] Wu S , Huang J, Dong J, Pan D. 2003. hippo encodes a Ste-20 family protein kinase that restricts cell proliferation and promotes apoptosis in conjunction with Salvador and Warts. Cell. 114:445–456. 12941273 10.1016/s0092-8674(03)00549-x

[jkab305-B85] Xie T , Spradling AC. 1998. decapentaplegic is essential for the maintenance and division of germline stem cells in the Drosophila ovary. Cell. 94:251–260. 9695953 10.1016/s0092-8674(00)81424-5

[jkab305-B86] Yatsenko AS , Shcherbata HR. 2021. Distant activation of Notch signaling induces stem cell niche assembly. PLoS Genet. 17:e1009489. 33780456 10.1371/journal.pgen.1009489 PMC8031783

[jkab305-B87] Yu G , Wang LG, Han Y, He QY. 2012. clusterProfiler: an R package for comparing biological themes among gene clusters. OMICS. 16:284–287. 22455463 10.1089/omi.2011.0118 PMC3339379

[jkab305-B88] Yuan S , Yu X, Topf M, Dorstyn L, Kumar S, et al 2011. Structure of the Drosophila apoptosome at 6.9 a resolution. Structure. 19:128–140. 21220123 10.1016/j.str.2010.10.009 PMC3053581

[jkab305-B89] Zheng Y , Pan D. 2019. The Hippo signaling pathway in development and disease. Dev Cell. 50:264–282. 31386861 10.1016/j.devcel.2019.06.003 PMC6748048

